# Ethnic disparities in complete and partial molar pregnancy incidence: a retrospective analysis of arab and jewish women in single medical center

**DOI:** 10.1186/s12889-024-18276-5

**Published:** 2024-05-29

**Authors:** Ala Aiob, Dina Gumin, Inna Zilberfarb, Karina Naskovica, Inshirah Sgayer, Susana Mustafa Mikhail, Avishalom Sharon, Lior Lowenstein

**Affiliations:** 1 Department of Obstetrics and Gynecology, Galilee Medical Center, POB 21, 22100 Nahariya, Israel; 2 Department of Obstetrics and Gynecology, Azrieli Faculty of Medicine, Bar Ilan University, Safed, Israel

**Keywords:** Molar pregnancy, Ethnicity, Complete mole, Partial mole, Arab, Jewish

## Abstract

**Background:**

Molar pregnancies, encompassing complete and partial moles, represent a rare and enigmatic gestational disorder with potential ethnic variations in incidence. This study aimed to investigate relations of ethnicity with risks of complete and partial molar pregnancies within an Israeli population while accounting for age differences.

**Methods:**

A retrospective study was conducted of data recorded during 2007–2021 in an academic medical center in Israel. The study population comprised 167 women diagnosed with complete or partial moles, for whom data were obtained through histological examination and P57 immunostaining. Maternal age and ethnicity were extracted from electronic medical records. Incidence rates were calculated per 10,000 live births, and a nested case-control study compared demographic characteristics and molar pregnancy incidences between Arab and Jewish women. Statistical analyses included age-adjusted comparisons, relative risk calculations and multivariate logistic regression.

**Results:**

The overall risk of molar pregnancy was 22 per 10,000 live births (95% confidence interval [CI] 18–25). Among Arab women, the overall risk was 21 (95% CI 17–25), and for PM and CM: 14 (95% CI 11–17) and 7 (95% CI 5–10), respectively. Among Jewish women, the overall risk was 23 (95% CI 18–29), and for PM and CM: 12 (95% CI 8–17) and 11 (95% CI 7–16), respectively. Among Arab women compared to Jewish women, the proportion of all the partial moles was higher: (65.3% vs. 51.6%, *p* = 0.05). The incidence of partial mole was higher among Arab than Jewish women, aged 35–39 years (26 vs. 8 per 10,000, *p* = 0.041), and did not differ in other age groups. After adjusting for age, the relative risk of partial moles was lower among Jews than Arabs (0.7, 95% CI 0.4-1.0, *p* = 0.053). For Arab compared to Jewish women, the mean age at molar pregnancies was younger: 31.0 vs. 35.1 years. However, other factors did not differ significantly between Arab and Jewish women with molar pregnancies. In multivariate analysis, Jewish ethnicity was significantly associated with a higher risk of complete molar pregnancies (OR = 2.19, 95% CI 1.09–4.41, *p* = 0.028).

**Conclusion:**

This study highlights ethnic differences in molar pregnancy risk within the Israeli population. Jewish ethnicity was associated with a higher risk of complete molar pregnancies, while Arab women had a significantly higher risk of partial moles. These findings underscore the need to consider ethnicity when studying gestational disorders. Further research should seek to elucidate the underlying factors contributing to these differences.

## Background

Molar pregnancy is a rare and enigmatic gestational disorder that is classified into two distinct entities: complete mole (CM) and partial mole (PM). These classifications are rooted in differences in gross morphology, histopathology and genetic characteristics [1–3]. A CM arises from either monospermic or dispermic fertilization of an anuclear oocyte, and is characterized by the absence of fetal tissue and marked trophoblastic hyperplasia [3, 4]. Conversely, a PM results from dispermous fertilization of a normal ovum; and manifests focal trophoblastic hyperplasia, villous swelling and the presence of identifiable fetal tissue [1–5]. Despite the intricate classifications and increasing awareness, the incidence of molar pregnancy demonstrates substantial geographic variation. This raises the potential of underlying factors such as genetics, nutrition, socioeconomic status and methodological discrepancies, as have been described in previous studies [6–8].

The role of race and ethnicity in molar pregnancy risk remains an area of limited understanding. While early investigations noted an increased prevalence among Asian women residing in Hawaii [10], other studies have yielded inconclusive results regarding ethnic associations [9, 11]. Furthermore, data about black and Hispanic women are notably sparse [12–15]. Notably, many of the relevant studies were conducted before the availability of advanced diagnostic tools, such as flow cytometry and immunohistochemical staining for p57. These tools have revolutionized diagnosing CM and PM accurately, especially in instances of equivocal histological findings [16–18].

In light of the abovementioned complexities and gaps in knowledge, we embarked on a rigorous hospital-based retrospective study within the confines of an academic medical center in Israel. Our primary objective was to explore the potential relations of ethnicity with the risks of CM and PM pregnancies. Given the well-established association between maternal age and molar pregnancy risk [19–21], we meticulously conducted an age-adjusted analysis to mitigate the confounding effects of variations in the age distribution of pregnant women across ethnicity groups.

## Methods

Before commencing this research, we received approval from the Institutional Review Board (Helsinki Committee) of Galilee Medical Center and the Israeli Ministry of Health (authorization number 0147-21-NHR in 01/02/2022).

The study population encompassed all the women diagnosed during 2007–2021 with CM or PM at the Obstetrics and Gynecologic Department of Galilee Medical Center, Israel, a regional medical center. In all instances of surgical uterine evacuation, whether due to spontaneous or induced abortion, the products of conception underwent histological examination. The diagnoses were established by pathologists at our institution based on histopathological features. P57 immunostaining was performed in all samples of hydatidiform mole (HM). We excluded from the analysis women who were of another ethnicity or who were referred to our center due to persistent elevation of human chorionic gonadotropin or for treating gestational trophoblastic neoplasia. Molar pregnancies that occurred in conjunction with twin pregnancies were also excluded. To calculate the incidence of molar pregnancy per live birth, we identified in our hospital obstetrical database all the singleton live births that occurred during 2007–2021. Maternal age and ethnicity data were extracted from electronic medical records, with ethnicity categories defined as Arab, Jewish and another.

The primary aim of this retrospective cohort study was to determine the incidence of molar pregnancies in our population, focusing on live births over a specified period. The data accessed from medical files, encompassing an examination of demographic characteristics, gravidity, parity, pathology reports, ultrasonographic reports, and laboratory data for all the women included. The incidence of hydatidiform moles was calculated per the total number of deliveries at our institution during the study period. Molar pregnancies identified within the specified timeframe were juxtaposed against the total number of live births recorded. This enabled calculating molar pregnancy incidence rates per a specific unit of the population (e.g., per 10,000 live births).

In a nested case-control study, our aim was to assess potential risk factors associated with molar pregnancies, and to compare between Arab and Jewish women. In particular, we compared between these groups, demographic characteristics, including age distribution (< 20 years, 20–24 years, 25–29 years, 30–34 years, 35–39 years, and 40 years and over), and incidences of CM and PM. Ethnicity-specific risk estimates of CM and PM per 10,000 live births were calculated, accompanied by corresponding 95% Poisson confidence intervals (CIs). This facilitated assessing both the incidence and risk factors associated with molar pregnancies within the study population.

Statistical analysis: Categorical data are summarized using frequencies and percentages, and continuous variables with normal distributions are presented as means ± standard deviations. We reported median values and ranges for variables not adhering to the standard distribution assumption. We employed the chi-square or Fisher’s exact test to compare categorical variables between groups when the expected frequency was less than 5. To compare continuous variables between groups, we used the independent t-test when the data displayed a normal distribution, as determined by a histogram of the variable’s distribution shape. A significance level of *P* < 0.05 was deemed statistically significant. Categorical and ordinal variables were compared using the χ2 test. Crude and age-adjusted relative risks (RR) for CM and PM were estimated using univariate and multivariate logistic regression, respectively. The age group was included as a covariate in the multivariate analysis. Women of unknown/other races were included in the models, but the results were not reported due to their limited numbers and heterogeneous nature. Odds ratios (ORs) were assumed to be a suitable approximation of relative risk for these uncommon outcomes. The referent group for all the calculations of RR was the group of Arab women, given its larger size. We conducted all the statistical analyses using SPSS Version 27.0 software.

## Results

We identified 167 women who were followed for a molar pregnancy during the study period. Of them, 101 had PM and 66 had CM; 101 were Arab women, 64 were Jewish, and two were of another ethnicity. During the study period, 78,665 singleton live births were recorded.

The risk of any molar pregnancy (CM and PM) for the entire population was 22 per 10,000 live births (95% confidence interval [CI] 18–25). The risk of a CM was 9 per 10,000 live births (95% CI 7–11); and for a PM, 13 per 10,000 live births (95% CI 11–16). Among Arab women, the risk of any molar pregnancy (CM or PM) was 21 per 10,000 live births (95% CI 17–25). The risks of PM and CM were 14 (95% CI 11–17) and 7 (95% CI 5–10), respectively, per 10,000 live births. Among Jewish women, the risk of any molar pregnancy was 23 per 10,000 live births (95% CI 18–29). The risks of PM and CM were 12 (95% CI 8–17) and 11 (95% CI 7–16), respectively, per 10,000 live births.

Tables 1 and 2 compare the incidences of CM and PM between Arab and Jewish women according to six age groups (< 20 years, 20–24 years, 25–29 years, 30–34 years, 35–39 years, and 40 years and over). The incidence of PM was significantly higher among Arab than Jewish women in the age group of 35–39 years (26 vs. 8 per 10,000, *p* = 0.041), while no differences were observed in other age groups. The incidence of CM was higher among Jewish than Arab women in the age group of 25–29 years, although this difference only reached marginal significance (10 vs. 4 per 10,000, *p* = 0.083, one-sided 0.054).

**Table 1:** Incidences of partial moles among Arab and Jewish women according to age groups

	Jewish			Arab			
Age (years)	Partial mole (N)	Total labors (N)	Partial mole per 10,000 live births	Partial mole (N)	Total labors (N)	Partial mole per 10,000 live births	P value 2-Sided
**< 20**	0	192	0	2	1569	13	**1.000
**20–24**	0	2793	0	12	13,794	9	**0.238
**25–29**	8	8385	10	13	17,054	8	*0.645
**30–34**	13	9625	14	15	10,665	14	*1.000
**35–39**	4	5233	8	12	4632	26	*0.041
**40>**	8	1269	63	12	980	122	*0.174

*Chi-Square Test, **Fisher’s Test

**Table 2:** Incidences of complete moles among Arab and Jewish women according to age groups

	Jewish			Arab			
Age (years)	Complete mole (N)	Labors (N)	Complete mole per 10,000 live births	Complete Mole (N)	Labors (N)	Complete mole per 10,000 live births	P value 2-Sided
**< 20**	0	192	0	3	1569	19	**1.000
**20–24**	2	2793	7	10	13,794	7	**1.000
**25–29**	8	8385	10	6	17,054	4	**0.083 #0.054
**30–34**	4	9625	4	7	10,665	7	*0.555
**35–39**	7	5233	13	4	4632	9	*0.557
**40>**	9	1269	71	5	980	51	*0.601

*Chi-Square Test, **Fisher’s Test, # 1-Sided

Crude and age-adjusted relative risks (RRs) for CM and PM (considering Arab women as the reference group) are presented in Table 3. After adjustment for age, Jewish compared to Arab women were less likely to have a PM (RR 0.7, 95% CI 0.4-1.0, *p* = 0.053). Compared to Arab women, Jewish women did not have a significantly higher risk of a CM (RR 1.3, 95% CI 0.8–2.2, *p* = 0.275).

**Table 3:** Crude and age-adjusted relative risks for complete mole and partial mole

	Crude relative risk (95% CI)	P value	Adjusted relative risk for age (95% CI)	Adjusted P value
**Complete mole**				
**Arab**	Referent		Referent	
**Jew**	1.5 (0.9–2.5)	0.094^*^	1.3 (0.8–2.2)	0.275^*^
**Partial mole**				
**Arab**	Referent		Referent	
**Jew**	0.9 (0.6–1.3)	0.602^*^	0.7 (0.4-1.0)	0.053^*^

^*^Chi-Square Test

Table 4 presents the characteristics of Arab and Jewish women who had molar pregnancies (CM and PM). The mean age of the Arab women with molar pregnancies was younger than that of the Jewish women: (31.0 vs. 35.1 years, *p* < 0.001). Among the Arab women, 65.3% of the molar pregnancies were PM and 34.7% were CM. Among the Jewish women, 51.6% of the molar pregnancies were PM and 48.4% were CM.

**Table 4:** The characteristics of Arab and Jewish women who had molar pregnancies

*N* = 167 Molar pregnancy	Total population	Jewish *N* = 64 (38.3%)	Arab *N* = 101 (60.5%)	P value
**Age, mean (range)**	32.0 (18–53)	35.1 (23–53)	31.0 (18–49)	**< 0.001** ^**^**^
**Partial mole, N (%)**	101 (60.5%)	33 (51.6%)	66 (65.3%)	**0.05** ^**#**^ 0.10^*^
**Complete mole, N (%)**	66 (39.5%)	31 (48.4%)	35 (34.7%)	**0.05** ^**#**^ 0.10^*^
**BMI (kg/m2), mean (± std)**	25.5 (± 5.1)	26 (± 5.8)	25 (± 4.6)	0.495^^^
**Smoking, N (%)**	11 (6.6%)	6 (9.4%)	4 (4.0%)	0.310^**^
**Regular menstruation, N (%)**	144 (86.2%)	51 (79.7%)	91 (90.1%)	0.386^*^
**Gestational age by LMP, mean (± std)**	9.7 (± 2.4)	9.4 (± 2.1)	10 (± 2.5)	0.146^^^
**Gestational age**				
**< 6 Weeks by CRL**	106 (63.5%)	41 (64.1%)	65 (64.4%)	0.635^*^
**6–9 Weeks by CRL**	47 (28.1%)	16 (25.0%)	29 (28.7%)	
**> 9 Weeks by CRL**	14 (8.4%)	7 (10.7%)	7 (6.9%)	
**Gravity, median (range)**	3 (1–11)	3 (1–11)	3 (1–10)	0.104^***^
**Parity, median (range)**	2 (0–7)	2 (0–7)	1 (0–6)	0.343^***^
**Abortions, median (range)**	0 (0–5)	0 (0–5)	0 (0–5)	0.263^***^
**Mole in the past, N (%)**	3 (1.8%)	2 (3.1%)	1 (1.0%)	0.560^**^
**IVF, N (%)**	2 (1.2%)	1 (1.6%)	1 (1.0%)	1.000^**^
**Beta-HCG**				
**< 50 K, N (%)**	34 (28.6%)	10 (22.7%)	24 (32.4%)	0.353^*^
**50-100 K, N (%)**	32 (26.9%)	11 (25.0%)	21 (28.4%)	
**> 100 K, N (%)**	53 (44.5%)	23 (52.3%)	29 (39.2%)	
**Blood type**				
**A+, N (%)**	63 (38.7%)	26 (41.9%)	37 (37.4%)	0.484^**^
**O+, N (%)**	46 (28.2%)	14 (22.6%)	30 (30.3%)	
**B+, N (%)**	27 (16.6%)	13 (21.0%)	14 (14.1%)	
**AB+, N (%)**	7 (4.3%)	2 (3.2%)	5 (5.1%)	

^t-test, ^#^one sided, *Chi-Square Test, **Fisher’s Test, *** Mann-Whitney.

LMP last menstrual period; CRL, crown-rump length; IVF, in vitro fertilization; HCG, human chorionic gonadotropin

The gestational age at presentation with molar pregnancies did not differ between the Arab and Jewish women (9.4 ± 2.1 vs. 10 ± 2.5 weeks, *p* = 0.146). Neither did the groups differ in BMI, smoking habits, gravity, parity, history of abortions, or history of molar pregnancies. Beta human chorionic gonadotropin levels were also similar at presentation.

In multivariate analysis, considering mole type as the dependent variable, and ethnicity and age as independent variables, ethnicity was significantly associated with the risk of a CM pregnancy. The risk of a CM was higher among Jewish than Arab women (OR = 2.19, 95% CI 1.09–4.41, *p* = 0.028).

## Discussion

In this retrospective single-center study, the overall risk of molar pregnancies was 22 per 10,000 live births, with a breakdown of 9 for CM and 13 for PM. We identified differences between Arab and Jewish women in incidences of molar pregnancies, according to the type of molar pregnancy and age groups. The mean age was younger for Arab than Jewish women (31 vs. 35 years).

The incidence of PM was higher among Arab than Jewish women, in the 35-39-year age group. The incidence of CM moles was higher among Jewish than Arab women, in the 25-29-year age group, but the statistical significance of this difference was only marginal. After adjusting for age, Arab women had a significantly higher risk of PM. In multivariable analysis, the risk of a CM was more than two times higher among Jews than Arabs.

The incidence of molar pregnancy has been shown to vary across studies, including some that reported rates similar to ours. For example, Melamed et al. reported an incidence rate of 24 per 10,000 live births for any molar pregnancy, and 13 and 11 per 10,000 live births for CM and PM, respectively [22]. Others reported higher [6] or lower rates; 14 and 12 moles per 10,000 live births were reported in the Netherlands and Sweden, respectively [7, 23]. These variations might be due to differences in population demographics, study methodologies and diagnostic criteria.

Regarding the younger age of Arab compared to Jewish women with molar pregnancies, we suggest that this may be due to cultural and social differences, including marriages at an early age among Arabs. This contrasts with a U.S. study by Lurain et al. [25] that did not find significant differences in the age distribution of molar pregnancies between ethnic groups (Caucasian and African American). Taken together, the data suggest that the ethnic differences in age distribution observed in our study may be specific to the Arab and Jewish populations under investigation.

We report differences between Arabs and Jews in mole types. Arab women tended to have a higher proportion of PM (65.3%), while Jewish women tended to have a higher proportion of CM (51.6%). Though these differences were marginally significant, they could indicate a possible role of ethnic background in the type of molar pregnancy.

The known correlation between molar pregnancy and age led us to perform an age-adjusted analysis and to compare Arabs and Jews. We identified significant differences between Arabs and Jews in the incidences of molar pregnancies across age groups. Specifically, for the ages 35–39 years, the incidence of PM was higher among Arab women; while for ages 25–29 years, the incidence of CM was higher among Jewish women. In a crude analysis, Jewish women had a higher risk of CM than Arab women, but this difference did not persist after adjusting for age.However, after adjusting for age, Arab compared to Jewish women had a significantly higher risk of PM.

In a multivariate analysis that considered the mole type as the dependent variable, and ethnicity and age as independent variables, ethnicity was significantly associated with the risk of CM pregnancy. Jewish women had a higher risk of a CM than Arab women, OR = 2.19. These findings are consistent with studies that showed correlations between ethnicity and molar pregnancy incidence; Melamed et al. revealed ethnic disparities between women in the United States. Asian compared to white women exhibited a higher likelihood of developing CM but were less likely to develop PM. Blacks were less likely than whites to develop PM and marginally less likely to develop CM. Similarly, Hispanics were less likely than whites to develop both CM and PM [22].. However, other studies found no significant differences in the incidence of molar pregnancies between ethnic groups [14, 15]. These discrepancies may be due to variations in population characteristics and geographic regions.

To our knowledge, this is the first report to describe the difference in molar pregnancy incidence between Arab and Jewish women. Notably, the vast majority of women in the area of our medical center give birth and are treated there. Additionally, these two ethnic groups, Arab and Jewish Israelis, live in a relatively small defined geographic area in northern Israel (The Galilee); this can neutralize the geographic bias. Moreover, in all instances of surgical uterine evacuation for termination of pregnancy in our department, the product of conception was sent for histological evaluation for HM. This increases the accuracy of the incidence, although the reported rate approximates HM incidence. Nonetheless, the ideal incidence of HM should be calculated as a percentage of pregnancies and not only live births.

Certain limitations of our study should be acknowledged; foremost is its retrospective design. The study’s sample size and potential biases should be considered when interpreting the results. The marginally significant findings should be interpreted cautiously and definitive conclusions cannot be reached due to the relatively small sample size. Additional multicenter studies with larger sample sizes are needed. Notably, as our medical center is a regional referral center, the patient population may not represent the entire patient population. This raises the possibility of referral bias.

## Conclusion

Overall, our findings suggest a possible relation between ethnicity and the risk of molar pregnancies, particularly CM. Jewish women had a higher risk of a CM than Arab women. In parallel, Arab women had a higher risk of PM after age adjustment. However, it is important to note that while these findings are intriguing, further research with larger sample size is needed to explore the underlying factors that might contribute to these differences and to determine whether similar patterns exist in other populations.

## Data Availability

The datasets used and/or analysed during the current study are available from the corresponding author upon reasonable request.

## Abbreviations

CM: Complete mole
PM: Partial mole
HM: Hydatidiform mole
